# Post-Ionization Dynamics of the Polar Molecule OCS in Asymmetric Laser Fields

**DOI:** 10.3389/fchem.2022.859750

**Published:** 2022-04-08

**Authors:** Tomoyuki Endo, Karl Michael Ziems, Martin Richter, Friedrich G. Fröbel, Akiyoshi Hishikawa, Stefanie Gräfe, François Légaré, Heide Ibrahim

**Affiliations:** ^1^ Institut national de la recherche scientifique, Centre Énergie Matériaux et Télécommunications, Varennes, QC, Canada; ^2^ Kansai Photon Science Institute, National Institutes for Quantum Science and Technology, Kizugawa, Japan; ^3^ Institute of Physical Chemistry and Abbe Center of Photonics, Friedrich Schiller University Jena, Jena, Germany; ^4^ Max Planck School of Photonics, Jena, Germany; ^5^ Department of Chemistry, Graduate School of Science, Nagoya University, Nagoya, Japan; ^6^ Research Center for Materials Science, Nagoya University, Nagoya, Japan

**Keywords:** two-color laser field, coherent control experiment, post-ionization dynamics, real-time real-space time-dependent density functional theory, Coulomb explosion imaging

## Abstract

We have investigated the dissociation mechanisms of the prototypical heavy polar molecule OCS into the two break-up channels of the dication, OCS^2+^ → O^+^ + CS^+^ and OC^+^ + S^+^, in phase-locked two-color intense laser fields. The branching ratio of the breaking of the C–O and C–S bonds followed a pronounced 2*π*-oscillation with a modulation depth of 11%, depending on the relative phase of the two-color laser fields. The fragment ejection direction of both break-up channels reflects the anisotropy of the tunneling ionization rate, following a 2*π*-periodicity, as well. The two dissociation pathways in the C–S bond breaking channel show different phase dependencies of the fragment ejection direction, which are assigned to post-ionization dynamics. These observations, resulting from the excitation with asymmetric two-color intense laser fields, supported by state-of-the-art theoretical simulations, reveal the importance of post-ionization population dynamics in addition to tunneling ionization in the molecular fragmentation processes, even for heavy polar molecules.

## 1 Introduction

Coherent control of molecular dynamics in real-time is one of the ultimate goals of chemistry. One can envision the production of novel chemical substances and chemical reactions without undesired side-products. Coherent reaction control with photons such as the pump-dump technique (Tannor and Rice, 1985; Shen et al., 1999) adapting the field to the instantaneous dynamics (Malinovsky et al., 1997; Gräfe et al., 2005) or interference between reaction pathways (Brumer and Shapiro, 1986; Wang et al., 1996) have been demonstrated in the past decades. Though these methods are powerful to achieve desired chemical reactions, possible reaction outcomes are limited by the structures of the potential energy surface (PES) along the reaction coordinates.

The development of ultrashort laser techniques opened-up new paths for direct reaction control by tailored intense laser fields (Rabitz, 2006). In intense laser fields (typically $∼10^{14}$ W/cm^2^), the PESs of molecules are distorted due to strong light-matter interactions. By using feed-back loop optimization of the shape of the laser electric fields, previous studies have demonstrated coherent control of photo-chemical reactions such as selective bond scission (Assion et al., 1998; Bergt et al., 1999; Brixner et al., 2001; Levis et al., 2001; Cardoza et al., 2005), intramolecular cyclization (Kotur et al., 2009), and electron localization (Wells et al., 2009). However, the complex relationships between the optimized laser fields and the obtained products hinder understanding of the fundamental driving mechanisms. Alternatively, one can control a chemical reaction by directly addressing its spectral signature (Ibrahim et al., 2009a,b).

Recently, simple pulse shaping techniques based on breaking the inversion symmetry of an electric field have been employed instead of complex tailored laser fields. Carrier-envelope-phase (CEP) stabilized few-cycle laser fields and phase-locked two-color laser fields are widely used to investigate the underlying mechanisms of such reaction control (Alnaser and Litvinyuk, 2017). Control of the fragment ejection direction has been demonstrated for a wide range of *non-polar* (symmetric) molecules, from the simplest diatomic molecule H_2_ (D_2_) (Kling et al., 2006; Roudnev and Esry, 2007; Ray et al., 2009; Znakovskaya et al., 2012; Wanie et al., 2016), summarized in (Ibrahim et al., 2018), to poly-atomic hydrocarbons (Xie et al., 2012, 2014; Alnaser et al., 2014; Miura et al., 2014; Song et al., 2015; Doblhoff-Dier et al., 2016), the tri-atomic CO_2_ molecule (Endo et al., 2016, 2017), as well as CF_4_ as part of this special issue (Hasegawa et al., 2022). Several mechanisms have been proposed such as the interference of dissociation pathways for $H_{2}^{+}$ and $D_{2}^{+}$ (Kling et al., 2006; Roudnev and Esry, 2007; Ray et al., 2009; Znakovskaya et al., 2012; Wanie et al., 2016; Ibrahim et al., 2018), coherent superposition of vibrational states and electron recollisional excitation for $C_{2}H_{2}^{+}$ (Alnaser et al., 2014; Xie et al., 2014; Song et al., 2015; Doblhoff-Dier et al., 2016), laser induced bond-weakening for $C_{2}D_{2}^{+}$ (Miura et al., 2014), or potential deformation for ${CO}_{2}^{2+}$ (Sato et al., 2003; Endo et al., 2016, 2017). The important roles of post-ionization interactions such as the interference between ionic states and potential deformation in doubly charged states as well as the ionization step in the fragmentation processes of non-polar molecules have been discussed and clarified by using such simple asymmetric laser fields.

In the case of *polar* (asymmetric) molecules on the contrary, the fragment ejection direction in asymmetric laser fields has been exclusively explained by the anisotropy of the tunneling ionization rate, which is determined by the shape of the ionizing molecular orbital (usually the highest occupied molecular orbital, HOMO) and the molecule’s permanent dipole moment (Ohmura and Tachiya, 2008; Holmegaard et al., 2010; Dimitrovski et al., 2011; Li et al., 2011; Wu et al., 2012; Ohmura et al., 2014; Li et al., 2016; Wustelt et al., 2018; Yue et al., 2018; Endo et al., 2019; Ohmura et al., 2019), rather than by post-ionization interactions. Although post-ionization interactions should be important even for the dissociation of polar molecules, their contributions are generally buried under the strong anisotropy of the tunneling ionization rate. A deeper understanding of both the ionization step and post-ionization interactions is required to achieve flexible and mighty reaction control.

In the present study, we investigated the break-up processes of carbonyl sulfide, OCS, in phase-locked two-color intense laser fields by using the Coulomb explosion imaging technique (Wales et al., 2014; Endo et al., 2020; Zhao et al., 2021). OCS is the prototype of a heavy polar molecule (not containing hydrogen atoms) and has been widely investigated, including a molecular movie of its alignment (Karamatskos et al., 2019). In addition, we have demonstrated the applicability of reaction control based on post-ionization interactions to the dynamics of heavy atoms by using a simple pulse shaping technique. The effects of post-ionization interactions between polar molecules and phase-locked two-color intense laser fields, and the resulting molecular dynamics are discussed on the basis of the two dissociation channels OCS^2+^, OCS^2+^ → O^+^ + CS^+^/OC^+^ + S^+^, and compared to state-of-the-art theoretical simulations using real-time real-space time-dependent density functional theory (rtTDDFT) and semi-classical surface-hopping dynamics.

## 2 Methods

### 2.1 Experimental Section

**FIGURE 1:**
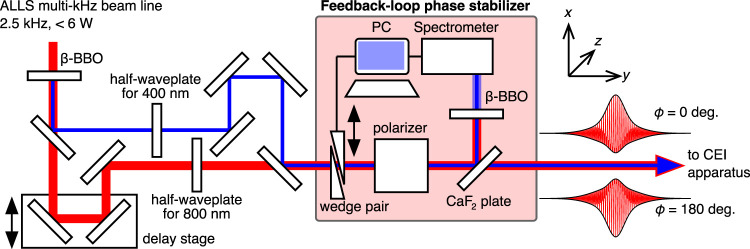
Schematic of the experimental setup to generate phase-locked two-color laser fields. The output of a Ti:Sapphire laser amplifier system (*ω*, 800 nm, 2.5 kHz) was introduced into a *β*-barium borate (BBO, Type I) crystal to generate second-harmonic pulses (2*ω*, 400 nm). *ω* and 2*ω* pulses were separated by a dielectric mirror. The polarization direction of both pulses was rotated by a half-waveplate and extracted by a polarizer to parallelize the polarization directions along the *x*-axis in the laboratory frame and tune the field intensity individually. The time-delay between both pulses was controlled by a linear delay stage before *ω* and 2*ω* pulses were collinearly re-combined. A pair of fused silica wedges was used to fine-tune the relative phase *ϕ*, which was locked by a feedback loop utilizing the interference spectrum of the 2*ω* pulses. Schematic shapes of the phase-locked two-color laser electric fields (*ϕ* =0° and 180°) are shown.

The 2.5 kHz Titanium-Sapphire laser beam line of the Advanced Laser Light Source (ALLS) user facility in Varennes, Canada, was used to perform ion coincidence three-dimensional (3D) momentum imaging measurements. Two-color laser fields were obtained in a Mach-Zehnder interferometer after second harmonic generation, as schematically shown in Figure 1.

The electric field of the resulting two-color laser field, composed of the fundamental (carrier frequency of *ω*) and parallel polarized second harmonic (2*ω*) pulses, can be expressed as

$$F\left(t\right)=F_{\omega }\cos\left(\omega t\right)+\;F_{2\omega }\cos\left(2\omega t+\phi \right)$$ (1)

where *F*_*ω*_ and *F*_2*ω*_ are the electric field amplitudes of the fundamental and second harmonic fields, respectively, and *ϕ* is the relative phase between both fields. The relative phase was determined keeping the fragment ejection direction consistent with previous OCS studies (Ohmura and Tachiya, 2008; Ohmura et al., 2014, 2019); *ϕ* is zero when the maximum amplitude side of *F*(*t*) points towards the positive *x* direction in the laboratory frame (see Figure 1).

To stabilize *ϕ* during long data acquisition times, the obtained two-color pulses were partly reflected by a CaF_2_ wedge. The reflected pulses were subsequently introduced into a second *β*-BBO crystal (Type I). The interference spectrum of both second harmonics generated by the first and the second BBO crystals was used to lock *ϕ* by a feedback loop with a pair of fused silica wedges mounted on a motorized translational stage (Endo et al., 2016, 2017). The root mean square of the measured phase deviation was less than 14°, corresponding to 0.05 fs over 24 h. The intensity of each laser field, $I_{\omega }\propto F_{\omega }^{2}$ and $I_{2\omega }\propto F_{2\omega }^{2}$ , at the focal spot was estimated by measuring pulse energy, pulse duration, and focal spot size of each beam. The pulse energy on target was estimated from the measured pulse energy in front of the chamber and the reflectivity of the chamber window and focussing mirror. The pulse durations of *ω* and 2*ω* pulses were measured to be 100 fs and 250 fs by second harmonic generation frequency-resolved optical gating (SHG-FROG) and transient grating FROG (TG-FROG) techniques, respectively (Kane and Trebino, 1993; Schmidt et al., 2008). The focal spot size was measured by a charge-coupled-device (CCD) camera using a second concave mirror of identical focal length in air. The total laser field peak intensity is defined as $I_{\omega +2\omega }\propto {\left(F_{\omega }+F_{2\omega }\right)}^{2}$ and the intensity ratio is defined as $\alpha =I_{2\omega }/I_{\omega }={\left(F_{2\omega }/F_{\omega }\right)}^{2}$ .

The obtained two-color laser fields were focused on an effusive molecular beam of a gas mixture (He 95% + OCS 5%) by a concave mirror (*f* = 100 mm) placed in an ultrahigh vacuum chamber. The generated ions were accelerated by electrodes in velocity map configuration to a position sensitive detector with delay-line anodes (RoentDek Handels GmbH). The 3D momentum of the *i*th ion $p_{i}=\left(p_{i}^{x},p_{i}^{y},p_{i}^{z}\right)$ was calculated from the position (*x*_*i*_, *y*_*i*_) and the arrival time (*t*_*i*_) at the detector. We used the Waterloo algorithm (Wales et al., 2012) to determine true coincidence events. The total kinetic energy release (KER) *E*_kin_ of each event was calculated from the 3D momenta as

$$E_{\text{kin}}=\underset{i}{\sum }\frac{{\left|p_{i}\right|}^{2}}{2m_{i}},$$ (2)

with *m*_*i*_ being the mass of the *i*th fragment ion.

### 2.2 Computational Section

Numerical simulations based on rtTDDFT and semi-classical surface-hopping dynamics were performed separately for the ionization and dissociation steps. First, we simulated the ionization of OCS in two-color laser fields under the fixed nuclei approximation, since the effects of bond stretching during ionization is not significant in the present experimental conditions. Second, to consider nuclear dynamics of OCS^2+^ in two-color laser fields, we simulated the dissociation processes starting from the selected electronic states.

#### 2.2.1 Ionization Step

We employed a state-of-the-art numerical description of the strong-field response of OCS (and OCS^+^) based on the rtTDDFT as realized in the *Octopus* program package (Andrade et al., 2012; Tancogne-Dejean et al., 2020). Shortly, the 16 outermost electrons, within the time-dependent Kohn–Sham orbitals, are propagated numerically in real-time and real-space using finite element methods. From these orbitals, the time-dependent electron density is constructed. For the calculations, a spherical box with a radius of *R* = 20 a. u. was used, including a complex absorbing potential at its boundary with *r*_abs_ = 5 a.u. We confirmed that increasing the box radius to *R* = 30 a. u. did not change the results substantially (a maximum deviation in the total number of emitted electrons Δ*n*_emit_ ∼ 0.002, <0.2% of the total number of emitted electrons *n*_emit_). We employed an equidistant grid of 0.28 a.u. resolution in three spatial dimensions each, resulting in more than 7,000,000 mesh points. The propagation is done in short (attosecond) time steps, requiring several ten-thousand consecutive propagation steps for covering a short excitation pulse of about 50 fs duration. Electron dynamics were propagated for > 150 fs (211,667 steps of about 0.7 as). The molecule is fixed in space and lies on the *x*-axis. The electric field is polarized likewise along the *x*-axis. The coordinate system is such that the carbon atom is at the origin, the oxygen at positive coordinate values, and the sulfur atom at negative ones ((*x*, *y*, *z*) positions: C (0,0,0); O (1.17,0,0); S (-1.57,0,0) a.u.).

For the exchange-correlation potential, we chose the local-density approximation (LDA) functional with average-density self-interaction correction (ADSIC). The highest occupied DFT Kohn–Sham orbitals included in our calculations can be specified as follows: the HOMO-5, HOMO-4, HOMO-3, and HOMO-2 are of *σ*-type, while the (doubly degenerate) HOMO-1 and HOMO are of *π*-type. We performed five different calculations for relative phases of *ϕ* = 0°, 45°, 90°, 135°, 180°. The laser pulses were implemented as in Eq. 1, with *F*_*ω*_ = 0.0755 a.u. (corresponding to an intensity *I*_*ω*_ = 2 × 10^14^ W/cm^2^), a pulse width of 20 fs, and a wavelength of 800 nm. For the 2*ω* field, a field strength of 0.0407 a.u. (corresponding to an intensity *I*_2*ω*_ = 0.29*I*_*ω*_ = 0.58 × 10^14^ W/cm^2^), a pulse width of 50 fs, and a wavelength of 400 nm are chosen. The pulse duration used in the simulation is shorter than that in the experiment due to the limitation of computational costs. However, as mentioned below in Section 3, since ionization mainly occurs around the peak of the laser electric field and dissociation from the excited state of OCS^2+^ proceeds in a few tens of femtoseconds, the pulse durations of 20 and 50 fs are long enough to compare with the experiment quantitatively. As the orbitals strongly mix in the presence of the laser field, we focus our evaluation on the projection of time-dependent Kohn–Sham wavefunctions on ground-state wave-functions at the end of the simulation time, when the pulse has vanished. By doing so, we can monitor the fraction of ionized electrons that originated from *σ* and *π* orbitals, respectively.

This procedure allows us to assign, which orbitals are depopulated in the field, and, consequently, which cationic (and dicationic) electronic states are formed upon ionization.

#### 2.2.2 Nuclear Dynamics of the Dication

The ground state equilibrium structure of neutral OCS was optimized by means of CASSCF(12, 10)/aug-cc-pVTZ level of theory (Aquilante et al., 2016) state-averaged over the first six singlet roots using the OpenMolcas suite program (Roos et al., 1980; Godbout et al., 1992). A subsequent vibrational analysis was carried out to verify that a minimum on the PES was obtained. The active orbitals were chosen by using the full set of valence orbitals (16 electrons in 12 orbitals) and omitting the two lowest *σ* orbitals with main contributions from 1*s* atomic orbitals of the oxygen and sulfur atom.

For the dynamical calculations of OCS^2+^, the semi-classical program package SHARC 2.1 (Richter et al., 2011; Mai et al., 2018) interfaced with OpenMolcas (Aquilante et al., 2016) was employed. The electronic structure properties were calculated using state-average CASSCF(10, 10)/cc-pVDZ for the first 20 roots in the triplet manifold, while the nuclei are treated classically. Non-adiabatic effects are realized in the SHARC code via jumps between electronic surfaces, and the two-color field is incorporated explicitly as off-diagonal element in the electronic Hamiltonian used for the propagation of the electronic wave function. The initial trajectories were Wigner sampled around the equilibrium structure (obtained as described above) of the neutral OCS and started in the triplet ground state of OCS^2+^, *X* ^3^Σ^−^, and the excited 1^3^Π state assuming an instantaneous excitation. For each of the three investigated relative phases of *ϕ* = 0°, 180°, 270°, an ensemble of 20 trajectories was started. The classic propagation was carried out for 75 fs with a time step of 0.1 fs centred around the light pulse. The KER was calculated by tracking the relevant mass-weighted molecular fragment in position space.

For all calculations, a reproducible and transferable computational environment was set up with the Nix package manager using NixOS-QChem (Kowalewski and Seeber, 2022) (commit 206dcba) and Nixpkgs (NixOS, 2021) (commit 9775b39).

## 3 Results and Discussion

**FIGURE 2:**
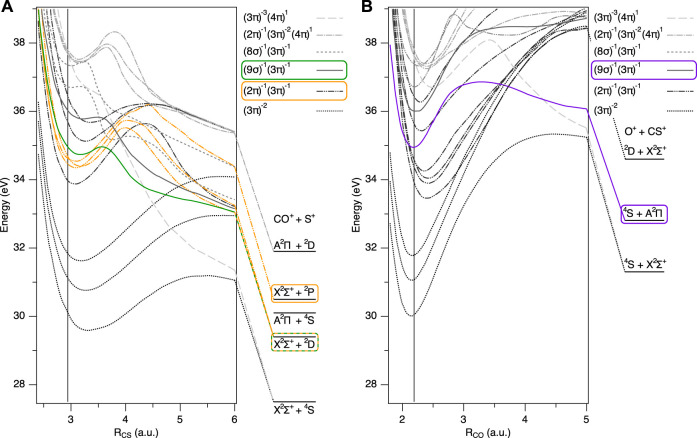
Potential energy curves of lower lying electronic states of OCS^2+^ and corresponding dissociation limits, dissociating to **(A)** OC^+^ + S^+^ (*R*_CO_ =2.185 a. u.) and **(B)** O^+^ + CS^+^ (*R*_CS_ =2.95 a. u.) (Brites et al., 2008). The bending angle is fixed at 180°. Vertical lines correspond to the equilibrium geometry of neutral OCS in the ground state. Dotted lines correspond to (3*π*)^−2^ states (*X* ^3^Σ^−^, *a* ^1^Δ, *b* ^1^Σ^+^), dash-double dotted lines correspond to (2*π*)^−1^(3*π*)^−1^ states (1^1^Σ^−^, 1^3^Δ, 1^1^Σ^+^, 2^3^Σ^−^, 2^1^Δ, 2^1^Σ^+^), solid lines correspond to (9*σ*)^−1^(3*π*)^−1^ states (1^3^Π, 1^1^Π), broken lines correspond to (8*σ*)^−1^(3*π*)^−1^ states (2^3^Π, 2^1^Π), dash-dotted lines correspond to (2*π*)^−1^(3*π*)^−2^(4*π*)^1^ states (2^1^Σ^−^, 2^3^Δ, 2^3^Σ^+^), and dashed lines correspond to (3*π*)^−3^(4*π*)^1^ states (1^5^Σ^−^). The latter two states include electron excitation to a vacant 4*π* orbital. The potential energy curves and corresponding dissociation limits mainly responsible for the KER peaks in Figure 3 are highlighted in green, orange, and purple.

The electronic configuration of neutral OCS in the ground state is (core)^14^(6*σ*)^2^(7*σ*)^2^(8*σ*)^2^ (9*σ*)^2^(2*π*)^4^(3*π*)^4^(4*π*)^0^. The potential energy curves of the lower lying electronic states, relevant to the two dissociation channels, OC^+^ + S^+^ and O^+^ + CS^+^, are depicted as a function of the bond lengths in Figure 2. The double ionization potential of OCS at the equilibrium structure (Franck-Condon region), reached by removing two valence electrons from the 3*π* orbital, is 30.3 eV (Langford et al., 1991). Since the electronic ground state of OCS^2+^ is the metastable *X* ^3^Σ^−^ state (Kaneyasu et al., 2015) and its lifetime is theoretically predicted to be on the order of 10^160^ s (Ridard et al., 1988), the dissociation does not occur directly from the ground state of OCS^2+^. In our nuclear dynamics simulation in intense laser fields, dissociation from the (3*π*)^−2^ states does not occur within our simulation time. Note that the molecular states are described in terms of single orbital occupations, which correspond to the dominant contributions.

As observed in the previous experiments of OCS in one-color intense laser fields (Bryan et al., 2006; Ma et al., 2019), OCS^2+^ preferentially dissociates into OC^+^ + S^+^ rather than O^+^ + CS^+^. The total yield of the OC^+^ + S^+^ channel is more than one order of magnitude larger than that of the O^+^ + CS^+^ channel in our experimental conditions. This can be explained by the fact that the products of the C–S bond breaking are more stable than the ones of the C–O bond breaking (Masuoka et al., 1992). The potential energies of the lowest state at the dissociation limits are 27.5 eV for OC^+^ (*X* ^2^Σ^+^) + S^+^ (^4^ *S*) vs 31.3 eV for O^+^ (^4^ *S*) + CS^+^ (*X* ^2^Σ^+^) (Brites et al., 2008). In addition, the potential barrier for the C–S bond breaking is much smaller than the one for the C–O bond breaking, as shown in Figure 2. According to photoionization experiments by Masuoka and Koyano (Masuoka and Koyano, 1991), the onset of the major OC^+^ + S^+^ dissociation channel is at a threshold energy of 33.5 eV, while more than 40 eV are necessary for the O^+^ + CS^+^ dissociation channel.

We start the discussion with the major dissociation channel, OCS^2+^ → OC^+^ + S^+^, compare it to previous studies of OCS in intense laser fields (Holmegaard et al., 2010; Dimitrovski et al., 2011; Ohmura et al., 2014, 2019; Ma et al., 2019; Zhao et al., 2021), and discuss its dissociation mechanisms. Next, we focus on the minor dissociation channel, OCS^2+^ → O^+^ + CS^+^. We then investigate the newly observed shoulder peak in the major dissociation channel and discuss the effects of post-ionization interactions. Lastly, we discuss the control of the branching ratio between the C–O and C–S bond breaking channels.

### 3.1 The Major Dissociation Channel: C–S Bond Breaking

**FIGURE 3:**
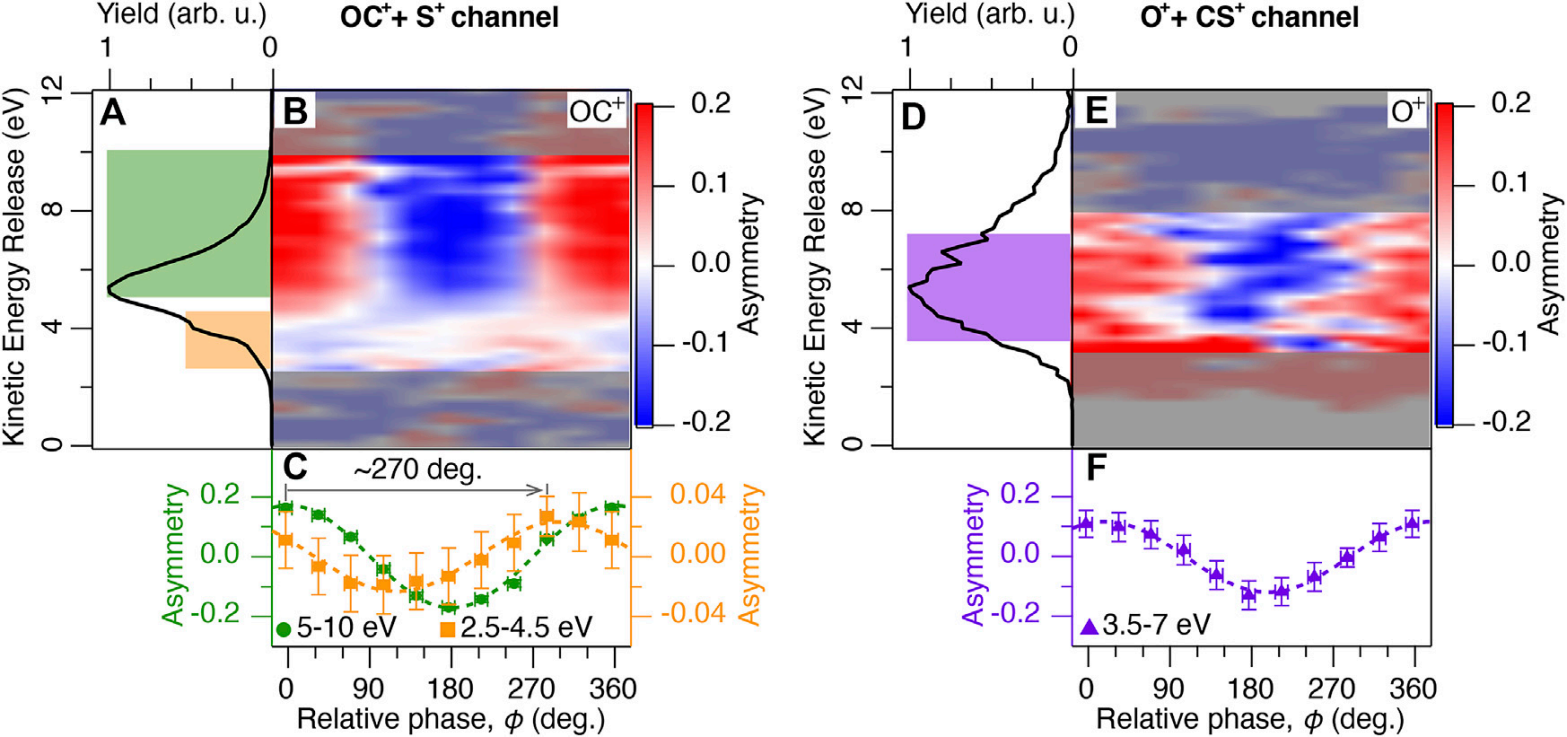
**(A)** The total kinetic energy release *E*_kin_ spectrum of the OC^+^ + S^+^ channel averaged over the relative phase *ϕ* between the *ω* and 2*ω* laser fields. The KER spectrum is calculated from the fragments ejected within 45 deg. with respect to the laser polarization direction. The colored areas are linked to the different dissociation pathways indicated in Figure 2. **(B)** Two-dimensional plot of the asymmetry parameter as a function of *E*_kin_ and *ϕ*. The asymmetry parameter is calculated from the OC^+^ fragment yields. Noisy data outside of the region of interest is masked grey shaded. **(C)** Asymmetry parameter of the OC^+^ fragments integrated over 5–10 eV (green circles) and 2.5–4.5 eV (orange squares) are plotted as a function of *ϕ*. The dotted lines are sine fits. **(D)** Same as **(A)**, but for the O^+^ + CS^+^ channel. **(E)** Same as **(B)**, but calculated from the O^+^ fragment yields. **(F)** Same as **(C)**, but the O^+^ fragments are integrated over 3.5–7 eV (purple triangles).

The phase-averaged total KER (*E*_kin_) spectrum of the OC^+^ + S^+^ channel in phase-locked two-color intense laser fields (*I*_*ω*+2*ω*_ = 2 × 10^14^ W/cm^2^, *α* = 0.19) is shown in Figure 3A. The *E*_kin_ spectrum shows a main peak at 5.2 eV (highlighted in green) and a shoulder structure at 4 eV (highlighted in orange).

The peak around 5 eV was also observed in a synchrotron experiment (Masuoka et al., 1992) and assigned to the fragments generated via the electronically excited states of OCS^2+^ such as (9*σ*)^−1^(3*π*)^−1^ or (2*π*)^−1^(3*π*)^−1^. Note that in a previous study using intense laser fields (Ma et al., 2019), the largest peak was however observed at a smaller KER of 4 eV instead of the 5.2 eV in our work. This difference can be attributed to the fact that different double ionization channels open up depending on the laser field intensity. In high intensity fields ( $\geq 5\times 10^{14}$ W/cm^2^), doubly charged OCS^2+^ ions would be produced by sequential double ionization (SDI), which involves bond stretching on the singly charged OCS^+^ ion, resulting in a smaller KER of 4 eV. In the present study, other mechanisms would be dominant due to the lower intensities.

**FIGURE 4:**
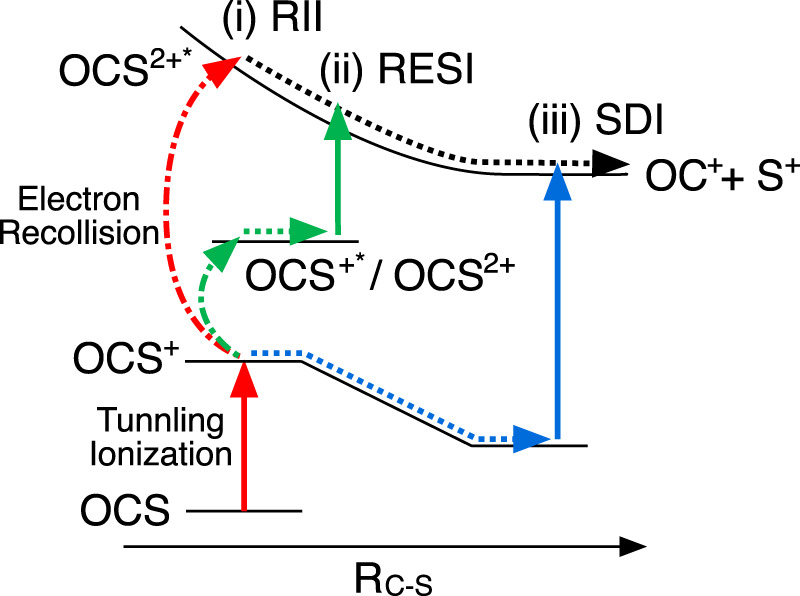
Schematic of sequential and non-sequential double ionization and dissociation processes of OCS along the C-S bond stretching coordinate. Three possible pathways for population to the excited states of OCS^2+^ (OCS^2+^*); (i) Recollision impact ionization (RII) leading to direct ionization to OCS^2+^* by electron recollision, (ii) recollisional excitation with subsequent ionization (RESI) causing indirect population to OCS^2+^* via intermediate states (OCS^+^* or OCS^2+^), and (iii) sequential double ionization (SDI) leading to ionization from OCS^+^, involving bond stretching. Due to the different bond stretching in the intermediate states and the shape of the PES of OCS^2+^*, the observed KERs appear in the order (i) > (ii) > (iii). Vertical solid arrows indicate ionization or excitation by laser fields. Curved dash-dotted arrows show ionization or excitation by electron recollision, dotted arrows represent bond stretching.

Three possible double ionization mechanisms in intense laser fields have been widely accepted, as indicated in Figure 4. One of them is SDI as mentioned above, others are identified as non-sequential double ionization (NSDI). SDI plays an important role in a higher intensity regime, because the first ionization step can occur on the rising edge of laser pulses and the residual laser fields are intense enough to remove the second electron by field ionization. However, in a lower intensity regime, the first ionization step occurs around the peak of the laser pulse. The residual field is not strong enough to lead to a second field ionization, therefore subsequent ionization is assisted by an electron recollision process. The simplest type of NSDI is recollision impact ionization (RII), in which the recolliding electron has gained sufficient energy to ionize the second electron. In the RII mechanism, since electron impact ionization occurs within half an optical cycle (1.3 fs at 800 nm) after the first ionization step, there is no time for bond stretching to occur. Another NSDI mechanism is known as recollision excitation with subsequent tunneling ionization (RESI), in which the recolliding electron can promote molecular ions to the excited states. The second electron will be subsequently ionized by the residual laser fields because of a smaller effective ionization potential. Due to the time delay between the first and second ionization step, the bond can slightly stretch in the RESI mechanism. Xu et al. (2020) clarified that NSDI is the dominant mechanism to generate OCS^2+^ at a laser field intensity below 1 × 10^14^ W/cm^2^ and SDI becomes dominant as the laser intensity increases.

In the present study, we observed the generation of fragments via the excited states of OCS^2+^ (OCS^2+^*) instead of the yields of the parent ion OCS^2+^. Therefore, the threshold intensity for SDI would be shifted towards larger intensity due to the larger potential energy of OCS^2+^* compared to the ground state of OCS^2+^. Here, three double ionization mechanisms, including the population of the excited state of OCS^2+^, should be considered as shown in Figure 4: i) direct ionization to the excited states of OCS^2+^* by electron recollision, that is RII to the excited states, ii) recollisional excitation (ionization) to OCS^+^* (OCS^2+^) with subsequent ionization (excitation) to OCS^2+^*, in analogy to RESI, and iii) SDI to the excited states of OCS^2+^.

**FIGURE 5:**
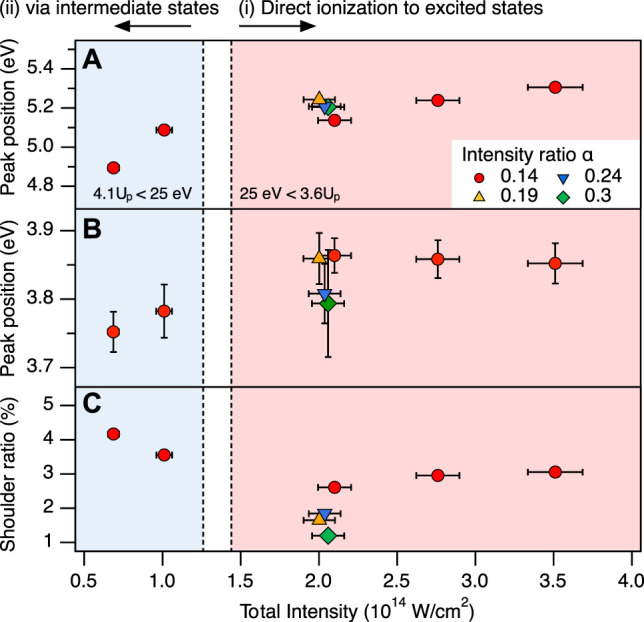
Intensity dependence of the KER spectrum. **(A)** Position of the main peak in the OC^+^ + S^+^ channel as a function of the total laser field intensity (*I*_*ω*+2*ω*_). **(B)** Position of the shoulder peak in the OC^+^ + S^+^ channel. **(C)** Ratio of the shoulder peak in the OC^+^ + S^+^ channel. Circles, triangles, up-side down triangles, and diamonds indicate the different intensity ratios of *α* = 0.14, 0.19, 0.24, and 0.3, respectively. Colored areas correspond to the intensity of (i) direct ionization to the 1^3^Π state of OCS^2+^ from the ground state of OCS^+^ by electron recollision (*I*_*ω*+2*ω*_ >1.44 × 10^14^ W/cm^2^) and (ii) indirect excitation via the electronically excited states of OCS^+^ or the electronic ground state of OCS^2+^ (*I*_*ω*+2*ω*_ <1.26 × 10^14^ W/cm^2^) in two-color laser fields at *α* = 0.14 (see the text for details).

To clarify contributions of these mechanisms, the laser field intensity dependence of the main peak position is shown in Figure 5A. We observe a larger peak shift in the lower field intensity region ( $<1\times 10^{14}$ W/cm^2^) than in the higher intensity region ( $>2\times 10^{14}$ W/cm^2^), which indicates switching of the major ionization mechanism from (ii) indirect via intermediate states to (i) direct ionization to the excited states between 1–2 × 10^14^ W/cm^2^. This switch is understood on the basis of the kinetic energy of the recolliding electron in intense laser fields. In two-color laser fields, this energy depends on the relative phase and the intensity ratio *α* as well as the laser field intensity (Endo et al., 2017). At *α* = 0.14, the maximum kinetic energy of the recolliding electron is between 3.6–4.1 *U*_p_ depending on the relative phase. Hereby, *U*_p_ is the ponderomotive energy in the fundamental laser fields and proportional to the laser field intensity. For example, the energy difference from the ground state of OCS^+^ to the excited 1^3^Π state of OCS^2+^, which has the smallest potential barrier along the C–S bond breaking, is about 25 eV at the equilibrium geometry of neutral OCS, so the corresponding intensity range is 1.26–1.44 × 10^14^ W/cm^2^. When the total laser field intensity is larger than the upper threshold, the maximum kinetic energy of the recolliding electron exceeds the vertical excitation energy regardless of the relative phase. In this intensity regime, the direct mechanism (RII to the excited states) contributes to populate the 1^3^Π states of OCS^2+^. As discussed above, the double ionization by RII occurs within half an optical cycle, which does not lead to distortion of the molecular geometry during ionization. On the other hand, at a total laser intensity below the lower threshold, the energy of the recolliding electron does not exceed the excitation energy. Therefore, the RII-type direct mechanism cannot contribute and RESI-type indirect mechanisms via the excited state of OCS^+^ or the ground state of OCS^2+^ play important roles. In the indirect mechanisms, bond stretching on intermediate states can occur, which may cause the lower KER. The observed KER shift in Figure 5A is well explained by this picture.

In addition, Wales et al. (2014) showed that the peak position of the KER spectra of the O^+^ + C^+^ + S^+^ channel from OCS^3+^ did not change with pulse duration below 200 fs; in contrast to the peak position from the OCS^4+^ break-up channel, which strongly depended on the pulse duration. The effects of bond stretching during ionization would be larger in higher charged states, because the bond can stretch at each charged state, causing further ionization of the molecules to an even higher charged state. Since the intensity of the fundamental pulse is much stronger than that of the second harmonic pulse in the present study, the pulse duration of the resulting two-color laser fields can be considered to be comparable to that of the fundamental pulse (100 fs). Bending is likely to occur under the present experimental condition, however, it would play a minor role in the two-body breaking channel we focused on in this study. Another concern might be the effect of photo-absorption of the 2*ω* field, due to its higher photon energy. We observe however, that the KER spectra in one-color and two-color laser fields show the same main and shoulder structure independent of *α* (see Supplementary Figure S3). Moreover, in longer wavelength (1700 nm + 850 nm) laser fields, similar main and shoulder peaks are observed at identical position compared to the shorter wavelength (800 nm + 400 nm). These results suggest that field ionization is the dominant mechanism and photo-absorption can be excluded.

From the discussion above and based on the PESs in Figure 2, we assign the main peak at 5.2 eV to dissociation via the electronically excited states 1^1^Π and 1^3^Π of OCS^2+^, populated by the RII mechanism. The electronic configuration of these states is (9*σ*)^−1^(3*π*)^−1^, leading to OC^+^ (*X* ^2^Σ^+^) and S^+^ (^2^ *D*) highlighted in green in Figure 2A. The states highlighted in Figure 2 represent the ones mainly contributing to the dynamics observed. We point out that, in general, for almost every triplet state, there is a corresponding singlet state of almost identical electronic configuration and similar fragmentation behavior (leading to almost identical KERs). Experimentally, we can not distinguish between singlet and triplet states, thus, also for the theoretical analysis, we concentrate on one spin species only (the triplet states). The shoulder peak is discussed in the separate Subsection 3.3.

**FIGURE 6:**
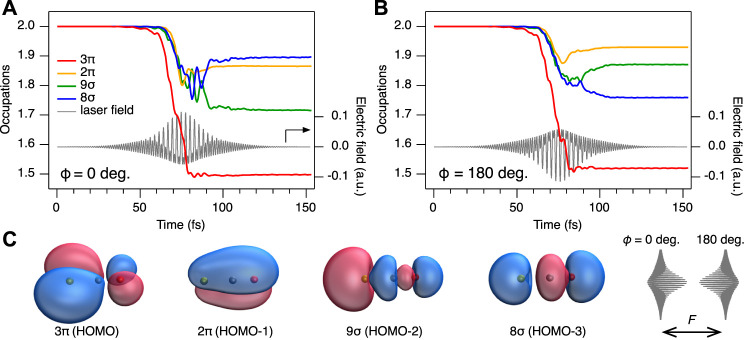
Results of the rtTDDFT calculations. **(A)** Time-dependent orbital occupations for *ϕ* = 0°. **(B)** same for *ϕ* = 180°. The temporal shapes of the electric fields are shown as solid grey lines. Note that 3*π* and 2*π* are doubly degenerate. **(C)** Orbitals of HOMO to HOMO-3. HOMO and HOMO-1 are *π* orbitals, while HOMO-2 and HOMO-3 are *σ* orbitals. For *π* orbitals, only one variant is shown, the other variant is a simple rotation around the molecular axis by 90°. Yellow atom indicate sulfur, blue atoms = carbon and red atoms = oxygen. *F* denotes the laser polarization direction. Schematic shapes of the phase-locked two-color laser electric fields (*ϕ* =0° and 180°) are shown.

To evaluate the fragment ejection direction quantitatively, the asymmetry parameter *A* is defined as a function of *E*_kin_ and *ϕ*,

$$A\left(E_{\text{kin}},\phi \right)=\frac{Y_{+}\left(E_{\text{kin}},\phi \right)-Y_{-}\left(E_{\text{kin}},\phi \right)}{Y_{+}\left(E_{\text{kin}},\phi \right)+Y_{-}\left(E_{\text{kin}},\phi \right)},$$ (3)

where *Y*_+_ and *Y*_−_ are the fragment yields with positive and negative momenta along the laser polarization direction (*x*-axis), respectively. Here, the fragment yields are evaluated by the counts of fragments ejected within 45° with respect to the laser polarization direction to reduce artificial blurring of the asymmetry due to the molecular rotation around the boundary of *Y*_+_ and *Y*_−_. The two-dimensional plot of the asymmetry parameter calculated from the OC^+^ fragment yields is shown in Figure 3B. A clear asymmetry was observed above 5 eV which showed a 2*π*-oscillatory behavior when changing the relative phase as observed in previous studies based on the fragments’ flight time measurements without coincidence detection (Ohmura et al., 2014, 2019). When a dissociation process is fast enough, the orientation of the parent ions generated by tunneling ionization reflects the ejection direction of fragment ions. Therefore, such strong asymmetry of the fragment ejection direction can be explained by the asymmetry of the ionization rate, which is determined by the shape of the HOMO and the effective ionization potential (Holmegaard et al., 2010). When the laser field is parallel to the difference in permanent dipole moment vectors of neutral and ionized states, the effective ionization potential becomes lower than in the field-free case and the ionization rate increases. Vice versa, in the case of an anti-parallel electric field, the ionization rate decreases since the effective ionization potential becomes higher than the field-free one. As a result of this competition between the shape of the HOMO and the effective ionization potential, the ionization rate increases when the laser field points to the O atom side and the electron ejects from the S atom side in circularly polarized laser fields (Holmegaard et al., 2010). However, in linearly polarized two-color laser fields (Ohmura et al., 2014, 2019), the effects of the permanent dipole moment are not strong enough to flip the ionization rate of OCS, as can be deduced from the shape of the HOMO (see Figure 6C), with larger electron density on the S atom than on the O atom side. That is, the ionization rate increases when the laser field points towards the O atom, and the electron ejects from the S atom side. Then, the OC^+^ fragments are preferentially ejected to the larger amplitude side of the two-color laser fields (positive direction of *x*-axis at *ϕ* = 0°).

### 3.2 The Minor Dissociation Channel: C–O Bond Breaking

The total KER spectrum of the O^+^ + CS^+^ channel is shown in Figure 3D. In contrast to the OC^+^ + S^+^ channel, no low-energy shoulder peak is observed. The two-dimensional plot of the asymmetry parameter calculated from the O^+^ fragment yields is shown in Figure 3E. No clear dependence on the KER is observed. The integrated asymmetry between 3.0 and 7.5 eV follows a similar phase dependence as the main peak of the OC^+^ + S^+^ channel, as shown in Figure 3F. Such similarity indicates that this broad peak can be assigned to the fragments originating from the excited 1^1^Π or 1^3^Π states. These states are the same ones contributing to the main peak of the OC^+^ + S^+^ channel, and dissociate to e.g. O^+^ (^4^ *S*) and CS^+^ (*A* ^2^Π) highlighted in purple in Figure 2B. The anisotropy of fragments from the excited 1^1^Π or 1^3^Π states reflects the angular dependence of the ionization rate because of the prompt dissociation through small potential barriers. Although the asymmetry amplitude slightly decreases compared to the main peak in the OC^+^ + S^+^ channel, which we attribute to the effects of molecular rotation due to a higher potential barrier along the O–C bond breaking. This result indicates that the orientation of the parent OCS^2+^ ions is mainly determined by the ionization step, even for different dissociation channels as discussed in the previous studies of polar molecules (Ohmura and Tachiya, 2008; Holmegaard et al., 2010; Dimitrovski et al., 2011; Li et al., 2011; Wu et al., 2012; Ohmura et al., 2014; Li et al., 2016; Wustelt et al., 2018; Yue et al., 2018; Endo et al., 2019; Ohmura et al., 2019).

### 3.3 Post-ionization Interaction: The Shoulder Peak in the Major Dissociation Channel

In the main OC^+^ + S^+^ dissociation channel, we have observed a shoulder peak at around 4 eV, next to the main peak, as indicated in orange in Figure 3A. This shoulder structure was not observed in synchrotron radiation experiments (Masuoka and Koyano, 1991; Masuoka et al., 1992). From the following discussion and nuclear dynamics simulation, we assign this peak to dissociation via the (2*π*)^−1^(3*π*)^−1^ states highlighted in orange in Figure 2A.

The peak position of the shoulder peak shifts in lower intensity region rather than in higher intensity region as well as the main peak. This shift indicates that the shoulder peak can be assigned to dissociation via the excited states of OCS^2+^, in a way similar to the main peak. To identify the origin of this shoulder peak from another point of view, we compare its ratio to the main peak depending on the laser field intensity in Figure 5C. From Xu et al. (2020) we know, that the SDI mechanism becomes dominant at higher intensities. If the shoulder peak can be attributed to fragments generated by the SDI process, its yield would significantly increase with increasing intensity. However, we observe that the yield in the OC^+^ + S^+^ channel remains almost constant as the total laser field increases. We therefore conclude that the occurrence of both the shoulder peak and the main peak are mainly caused by the RII mechanism that does not involve bond stretching.

Another possible pathway for the occurrence of the shoulder peak would be due to interactions in neutral states such as enhanced ionization induced by structural deformation. In general, enhanced ionization causes a decrease of the observed KER (Ibrahim et al., 2018); the main peak, however, appears at the same energy here and also in the case of a synchrotron experiment (Masuoka et al., 1992) and bond stretching in neutral states was not observed in previous studies (Wales et al., 2014; Ma et al., 2019). From these observations, we conclude that interactions in neutral states are not significant here and that the ionization process, vertical ionization in the Franck-Condon region, is essentially the same as in previous studies. However, interactions after ionization/during dissociation, such as potential deformation or population transfer play important roles in the dissociation of polar molecules, as we discuss in the next sections.

The shoulder peak is not only present, it also shows a different phase dependence in its asymmetry parameter *A*, compared to the main peak. Its asymmetry amplitude is about 5 times weaker, and the relative phase leading to maximum asymmetry is shifted by 270° (-90°). This shift is directly apparent in the integrated asymmetry parameters between 5 and 10 eV for the main peak and between 2.5 and 4.5 eV for the shoulder peak in Figure 3C. Such shift indicates that contributions from two (or more) components are present whose phase difference is not an integer multiple of 180°. We draw this conclusion, because two components, phase-shifted by 180°, would only cause a dependence of the overall asymmetry amplitude on their ratio but no phase shift would occur.

The weak amplitude can be attributed to competition between the contributions from the main and shoulder peaks with different phase dependence, leading to a cancellation in modulation contrast. The observed phase shift, however, cannot be explained by the simple tunneling ionization picture discussed above for the main peak. The ionization rate becomes largest at a relative phase of 0° or 180°, where the peak of the electric field amplitude is maximum. In the extreme case of diametrical effects of the orbital shapes and dipole moments, e.g. ionization from lower lying orbitals, the asymmetry would be shifted by 180°. The observed phase shift of 270° therefore means that other mechanisms, occurring after the actual ionization, such as electron recollisional excitation, potential deformation, or population transfer between electronic states determine the fragment’s asymmetry. One of the possible scenarios is based on electron recolliding excitation and potential deformation as suggested in the asymmetric dissociation of CO_2_ (Endo et al., 2017): The excitation probability due to electron impact depends on the kinetic energy and the incident direction of the recollisional electron, which are determined by both the field intensity and the relative phase (Endo et al., 2019). This means that the population distribution among electronic states in the ionic states manifold depends on the relative phase. In addition, potential deformations would be more prominent in higher electronic states, which have diffuse wavefunctions. The sum of these contributions from different electronic states can lead to the observed phase shift of 270° in the fragment ejection direction. However, in the case of CO_2_, the asymmetry amplitudes are on the order of a few percent, while in this study, they reach values of up to 0.16 (*E*_kin_ > 5 eV) and decrease to 0.03 (*E*_kin_ < 4.5 eV). This suggests that both shoulder- and main peak have comparable asymmetry amplitudes. In addition to that, the intensity dependence of the asymmetry amplitudes of CO_2_ (Endo et al., 2017) plateaus, while in OCS a monotonic decrease with increasing intensity is observed as shown in Supplementary Figure S4 of the SM. We therefore suggest another possible scenario; a population transfer between electronic states after ionization, as will be discussed later.

To summarize these results, the shoulder peak of the OC^+^ + S^+^ channel occurring around 4 eV exhibits a distinct phase dependence compared to the two channels directly dissociating from the excited states. We assign its origin to post-ionization dynamics; through this observed phase dependence, we are able to indicate that this shoulder peak arises from a population transfer between electronic excited states in doubly charged states, as supported by the computational results discussed in the following.

### 3.4 Computational Results

To further analyse the contribution of different electronic states to the observed asymmetry in both ionization and post-ionization interactions, we now discuss the computational results of the rtTDDFT calculations. Results are summarized in Table 1 and Supplementary Figure S1 of the SM, listing the total number of emitted electrons *n*_emit_, the number of electrons originating from 3*π* and 2*π* orbitals (HOMO and HOMO-1), *n*_*π*_, and from 9*σ* and 8*σ* orbitals (HOMO-2 and HOMO-3), *n*_*σ*_, as well as the fraction of electrons originating from *σ* orbitals, *f*_*σ*_ = *n*_*σ*_/*n*_emit_. The results show that for phase *ϕ* = 0°, ionization is most efficient: 1.67 electrons are removed in total. This is not surprising, as the peak of the field amplitude |*F*_*ω*+2*ω*_| is maximum for that phase. However, for a relative phase of *ϕ* = 180°, the ionization is less, as only 1.48 electrons, about 90% of *ϕ* = 0°, leave the numerical grid. Thus, as the molecule in the simulation is perfectly aligned with the laser polarization direction, at phase *ϕ* = 0°, ionization preferentially occurs from the sulfur atom, as the electric field vector pointing towards the oxygen atom allows the electrons to acquire momentum in the opposite direction. For phase *ϕ* = 180°, the situation is reversed and ionization preferentially occurs from the oxygen side. As shown by Ohmura et al. (Ohmura and Tachiya, 2008; Ohmura et al., 2014, 2019), the ionization efficiency is larger when emitting electrons from the large lobe of the 3*π* HOMO located at the sulfur (see Figure 6C), explaining the observed difference in *n*_emit_ for the cases *ϕ* = 0° and *ϕ* = 180°. Note, that the preference of ionization from the S or the O side at specific *ϕ* is identical between our work and the ones of Ohmura et al. (Ohmura and Tachiya, 2008; Ohmura et al., 2014, 2019), both obtained with linear polarized light. It is however opposite to the work of Holmegaard et al. (Holmegaard et al., 2010) using circularly polarized light.

**TABLE 1:** Summary over the numerical results obtained from the rtTDDFT calculations. Listed are the number *n*_emit_ of emitted electrons originating from 3*π* and 2*π* orbitals (HOMO and HOMO-1), *n*_*π*_, and from 9*σ* and 8*σ* orbitals (HOMO-2 and HOMO-3), *n*_*σ*_, as well as the fraction of electrons originating from *σ* orbitals, *f*_*σ*_, for different relative phases *ϕ*. Additionally, the maximum field strength of the (combined) applied laser field, |*F*_max_|, is given in atomic units (a.u.).

phase *ϕ*	0°	45°	90°	135°	180°
*n*_emit_	1.67	1.61	1.48	1.46	1.48
*n*_*π*_	1.27	1.25	1.16	1.11	1.10
*n*_*σ*_	0.40	0.36	0.32	0.34	0.38
*f*_*σ*_	0.24	0.23	0.22	0.24	0.26
|*F*_max_|	0.1157	0.1117	0.1004	0.1120	0.1160

Next, we consider the amount of *σ* electrons being emitted for different relative phases. Figure 6A,B provide an overview over the time-dependent occupations of the respective orbitals for *ϕ* = 0° and 180°. Overviews of other phases are provided in the SM. It should be noted that the *π* orbitals are doubly degenerate. We can read from Figure 6 and Table 1 that the number of electrons emitted from *σ* orbitals *n*_*σ*_ is 0.40 for *ϕ* = 0°, while *n*_*σ*_ = 0.38 for *ϕ* = 180°. Emission of *σ* electrons corresponds to a depopulation of the 9*σ* and 8*σ* orbitals, which leads, together with the also very pronounced depopulation of the 3*π* orbitals, to a final electron configuration of (9*σ*)^−1^(3*π*)^−1^ and (8*σ*)^−1^(3*π*)^−1^, and thus to the 1^1^Π, 1^3^Π, 2^1^Π, and 2^3^Π states. These states feature a small barrier towards dissociation into OC^+^ + S^+^ (Figure 2A). Thus, upon pronounced ionization of *σ* orbitals, the dication ultimately fragments into OC^+^ + S^+^. As a side notice, fragmentation of these electronic states into the O^+^ + CS^+^ channel is less likely, as the barrier towards dissociation is still around 2 eV (see also Figure 2B). This explains the difference of the yields between the channels observed in the experiment, and also why the main peaks at 5 eV in the two different fragment channels follow the same phase dependence (green line in Figure 3C and purple line in (f)).

The rtTDDFT calculations also provide another possible post-ionization origin to explain the shoulder peak in the OC^+^ + S^+^ channel: taking a closer look again on the population dynamics in Figure 6A, we observe a strong mixing between the 9*σ* and 8*σ* orbitals for *ϕ* = 0°. This can be traced back, because the 2^1^Π and 2^3^Π states (corresponding to ionization from 8*σ*) are, at the equilibrium structure of the neutral OCS, only about 2 eV above the 1^1^Π and 1^3^Π states (corresponding to ionization from 9*σ*, see Figure 2). As can be gathered from the time-dependent populations in Figure 6, mixing of these states starts around *t* = 70 fs and continues up to 100 fs, a time well after the maximum of the electric field and also after the main ionization event between *t* = 60–80 fs. This state mixing after the main ionization events provides another strong indication for post-ionization dynamics. Figure 2 shows that at slightly stretched geometries (*R* = 3.5 a. u.), the pairs of the two mixing states, 1^3^Π and 2^3^Π, or 1^1^Π and 2^1^Π, come energetically very close and the efficiency of population transfer can be expected to further increase at the wing of the pulse compared to the rtTDDFT calculations, which do not take such stretching into account. This is similar to the strong coupling of the *σ*_*g*_ and *σ*_*u*_ states in $H_{2}^{+}$ at a bond distance larger than the equilibrium geometry (Kling et al., 2006; Roudnev and Esry, 2007; Ray et al., 2009; Znakovskaya et al., 2012; Wanie et al., 2016; Ibrahim et al., 2018). This “late” population transfer is expected to lead to fragments with smaller kinetic energies, around 3–4 eV. Post-ionization interactions, such as electron recollisional excitation, potential deformation, and population transfer, would contribute to the shoulder peak and the competitions of these effects determine the effective phase dependence. We have therefore demonstrated here post-ionization interactions of heavy polar molecules in both experiment and calculation.

Since the rtTDDFT calculations already strongly suggested the contribution of several excited states, we performed separate semi-classical surface-hopping calculations, in order to trace possible post-ionization dynamics beyond the fixed-nuclei approximation. As outlined in Section 2.2.2, in these calculations, the electronic states are calculated on-the-fly with the quantum chemical program package OpenMolcas including the two-color laser field, while the nuclei are treated classically.

We have investigated the following initial conditions upon double ionization: 1) all trajectories are launched in the electronic ground state of the dication, *X* ^3^Σ^−^; 2) all trajectories start initially in the excited state 1^3^Π ((9*σ*)^−1^(3*π*)^−1^), as suggested by rtTDDFT.

**FIGURE 7:**
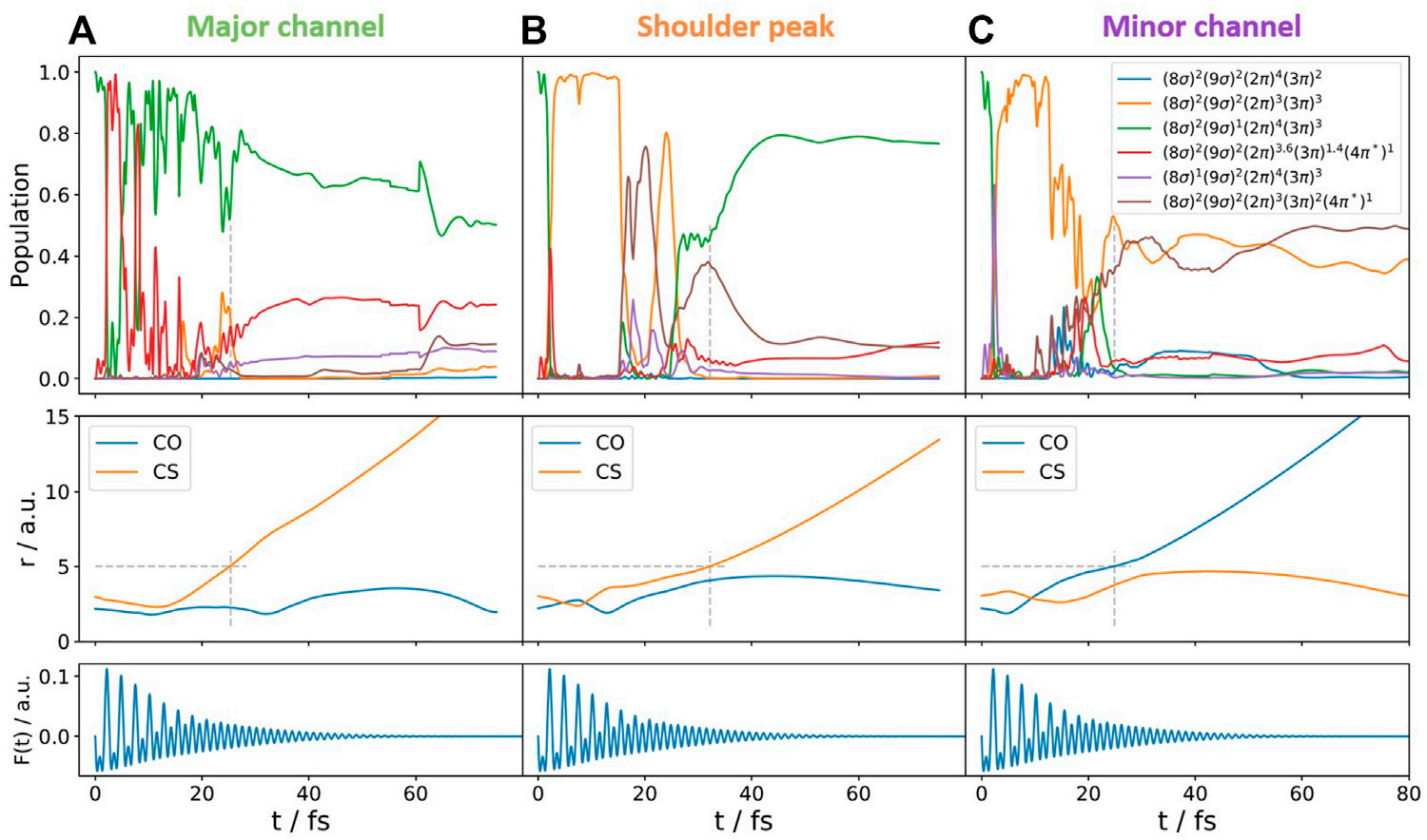
Quantum amplitudes (in the limit of infinite trajectories, this would correspond to populations) of different excited states (top), bond length C-O and C-S (middle), and the temporal shapes of the applied electric fields (bottom) during the dissociation of selected trajectories. These represent typical trajectories following **(A,B)** the OC^+^ + S^+^ channel and **(C)** the O^+^ + CS^+^ channel. A KER > 5 eV **(A)** is associated with the main peak of the OC^+^ + S^+^ channel in the experiment, while a KER < 4.5 eV **(B)** is attributed to the shoulder peak. The KERs at the end of these specific trajectories correspond to 7.9 eV **(A)**, 3.9 eV **(B)**, and 5.8 eV **(C)**. Dashed gray lines indicate the time when the (arbitrarily chosen) value of *r* = 5 a. u. is reached. We note that such dissociation is reached at a later time for the shoulder peak compared to the main peak; another strong indication for post-ionization dynamics.

Our calculations show that for (i), double ionization occurring to the electronic ground state of the dication, none of the trajectories showed dissociation during the calculation time. These results will not be discussed any more in what follows. For (ii), ionization into the excited electronic state of OCS^2+^*, several pathways were identified for a relative phase of *ϕ* = 0°, leading to break up into OC^+^ + S^+^ (4 of 20 trajectories), O^+^ + CS^+^ (5 of 20 trajectories), and [O+ C + S]^2+^ (11 of 20 trajectories), the latter one being not visible experimentally. Similar values concerning the relative number of trajectories are obtained for the other relative phases of the two-color field. When looking at the population dynamics, we gather a complex behavior for all cases, including population transfer between several excited electronic states (see also Figure 7 for representative trajectories). Changes in the relative phase of the two-color field lead to changes in the ratio between the population of the electronic states. With these trajectories at hand, we focus on the OC^+^ + S^+^ channel and the question concerning the shoulder peak. In our calculations, we identify for this break-up channel trajectories which would end up at higher KER ( > 5 eV), as well as a substantial number of trajectories ending up at lower KER ( < 5 eV). Figure 7 shows the population dynamics of two representative trajectories; panel A) displays the population dynamics of a trajectory leading to fragmentation at higher KER corresponding to the main peak of the OC^+^ + S^+^ channel. Panel B) shows the respective scenario for a trajectory leading to fragmentation at lower KER. Panel C) shows the population dynamics of a trajectory leading to the O^+^ + CS^+^ channel. We can see that the dissociation leading to higher KERs (main peak) proceeds either directly from the (9*σ*)^−1^(3*π*)^−1^ state (green line) or involves the mixed-character ${\left(2\pi \right)}^{-0.4}{\left(3\pi \right)}^{-2.6}{\left(4\pi^{*}\right)}^{1}$ state (red line), while the dissociation leading to lower KERs (shoulder peak) proceeds with pronounced coupling to the (2*π*)^−1^(3*π*)^−1^ state (orange line). Moreover, the dynamics involving population transfer seems to occur at later times. Or, in other words, the dynamics associated to the shoulder peak at low KER seems to proceed slower and it takes slightly longer to achieve the same C–S elongation. Thus, our semi-classical surface-hopping calculations emphasize the importance of post-ionization population dynamics.

### 3.5 Control of the Branching Ratio

**FIGURE 8:**
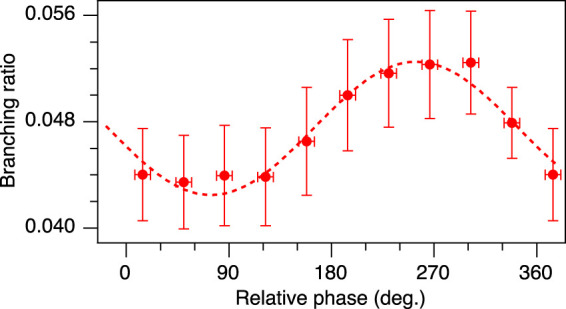
Inter-channel branching ratio between the OC^+^ and the O^+^ fragments ejected to the same direction as a function of the relative phase at a total peak intensity of 2×10^14^ W/cm^2^ and a ratio *α* = 0.14. Red circles correspond to experimental data points, the dotted line is a sine fit.

To discuss the effects of post-ionization interactions from another point of view, the inter-channel branching ratio between the OC^+^ and the O^+^ fragments is defined as

$$B\left(\phi \right)=\frac{Y_{+,{\text{O}}^{+}}\left(\phi \right)}{Y_{+,{\text{OC}}^{+}}\left(\phi \right)},$$ (4)

where $Y_{+,{\text{OC}}^{+}}$ and $Y_{+,{\text{O}}^{+}}$ are the OC^+^ and O^+^ fragment yields with positive momentum along the laser polarization direction, respectively. The fragment yields are evaluated in the same way as *Y*_+_ in Eq. (3), but integrated over the entire KER range. Those fragments were detected by ion-ion coincidence measurements, as in the previous sections. In other words, this parameter compares the yields of OC^+^ and the O^+^ fragments ejected to the same direction and evaluates whether the O–C or the C–S bond is preferentially broken in the dication. The branching ratio is shown as a function of the relative phase in Figure 8. The total peak intensity *I*_*ω*+2*ω*_ = 2 × 10^14^ W/cm^2^ is the same as in Figure 3, but the intensity ratio, *α* = 0.14, is smaller. The branching ratio shows a clear 2*π*-oscillation with a modulation depth of 11%. This oscillation indicates that the O–C bond-breaking is more likely to occur at 255° than at 75°. As discussed above, we consider NSDI to be the dominant process in the present experimental conditions. Moreover, we could exclude contributions of bond stretching in neutral states because such effects are not observed even in higher intensity laser fields (Ma et al., 2019). Therefore, the origin of this phase dependence can be attributed to post-ionization interaction rather than to the ionization step. Dissociation from the 2^3^Π state would enhance the C–S bond breaking due to a smaller potential barrier of the 2^3^Π state compared to the 1^3^Π state along this bond and almost similar potential barriers of these states along the C–O bond. Therefore, the C–S bond breaking is more likely to occur and the branching ratio decreases when population transfers from the 1^3^Π state to the 2^3^Π state or electron recollisional excitation to the 2^3^Π state occurs. In two-color laser fields, the recolliding electron energy has minimum and maximum values at relative phases of around 25° and 110°, so the population distribution in the doubly charged states would depend on the relative phase as well as on the dynamics after the ionization. In the rtTDDFT calculations, 8*σ* occupations, which correspond to populating the 2^3^Π state at the end of the simulation, show a clear phase dependence. Thus, this population transfer scenario can explain both the observation of the shoulder peak and the phase dependencies of the asymmetry parameters and the branching ratio.

We have thus demonstrated the selective bond scission of polar molecules by changing the relative phase of phase-locked two-color intense laser fields and attributed the origin of such selectivity to post-ionization interactions.

## 4 Conclusion

To summarize, we have investigated the Coulomb explosion of the polar molecule OCS in phase-locked two-color intense laser fields. A clear 2*π*-oscillation was observed in both the OC^+^ + S^+^ and O^+^ + CS^+^ channels. We have succeeded in the observation of a shoulder peak that has not been discovered previously, because it is a dark channel for synchrotron radiation that opens however up due to strong light-matter interactions in intense laser fields. In the OC^+^ + S^+^ channel, a clear dependency of the asymmetry on the kinetic energy was found due to this dissociation pathway. The phase shift of 270° between both sub-channels of OC^+^ + S^+^ is attributed to a population transfer between the involved electronic states in the applied intense laser fields as demonstrated by rtTDDFT calculations. In addition, the inter-channel branching ratio (when breaking the C–O and C–S bonds oriented to the same direction with respect to the laser polarization direction) also oscillated with 2*π*, depending on the relative phase. The selective scission of chemical bonds in polar molecules has been demonstrated by using a simple pulse shaping technique. Here presented results indicate that not only the ionization step, but also post-ionization interactions play important roles even for heavy polar molecules, where the anisotropy of the fragments is mainly dominated by tunneling ionization. Two-color phase-locked laser fields provide a variety of controllable parameters, such as the total intensity, the intensity ratio, the relative phase and the polarization, while not involving a complex experimental implementation. This approach is therefore useful to investigate the mechanisms of coherent reaction control for a wide variety of molecular systems by tailored intense laser fields.

## Data Availability

The raw data supporting the conclusions of this article will be made available by the authors, without undue reservation.
