# Supplementary material for: Post-Ionization Dynamics of the Polar Molecule OCS in Asymmetric Laser Fields
Source: Front Chem. 2022 Apr 8;10:859750. doi: 10.3389/fchem.2022.859750 (PMC9023801; doi:10.3389/fchem.2022.859750)
Supplement: Supplementary file 1 [file DataSheet1.pdf]

Supplementary Material

1 PHASE DEPENDENCE OF RTTDDFT RESULTS

The time-dependent occupations for the relative phases of $\phi = 45°, 90°, 135°$ are shown in Fig. S1. The orbital occupations at the end of the simulation depends on the relative phase (see Fig. S1(d)). In addition, the couplings between 9σ (HOMO-2) and 8σ (HOMO-3) between the time $t \sim 60 - 100$ fs, continuing after the main ionization event, vary the orbital occupations depending on the relative phase. This means that the electronic states after ionization are varied by post-ionization interactions as well as ionization step (e.g. which orbital electron emits from).

A note on the number of electrons reported in Table 1 of the main text and the observed different number of electrons emitted for almost identical field strength (1.67 electrons at ϕ = 0 and 1.48 electrons at ϕ = 180° : Assuming that the polarization would have no effect at all on the electron dynamics and only depend on e.g. the field strength, the difference should be almost zero (maximum field strength differs by about 1% only) and the maximum field strength is even higher at $\phi = 180°$ than at ϕ = 0°. However, what we see in electron numbers is a difference of about 11%, meaning that there is a clear dependence of ionization on laser polarization.

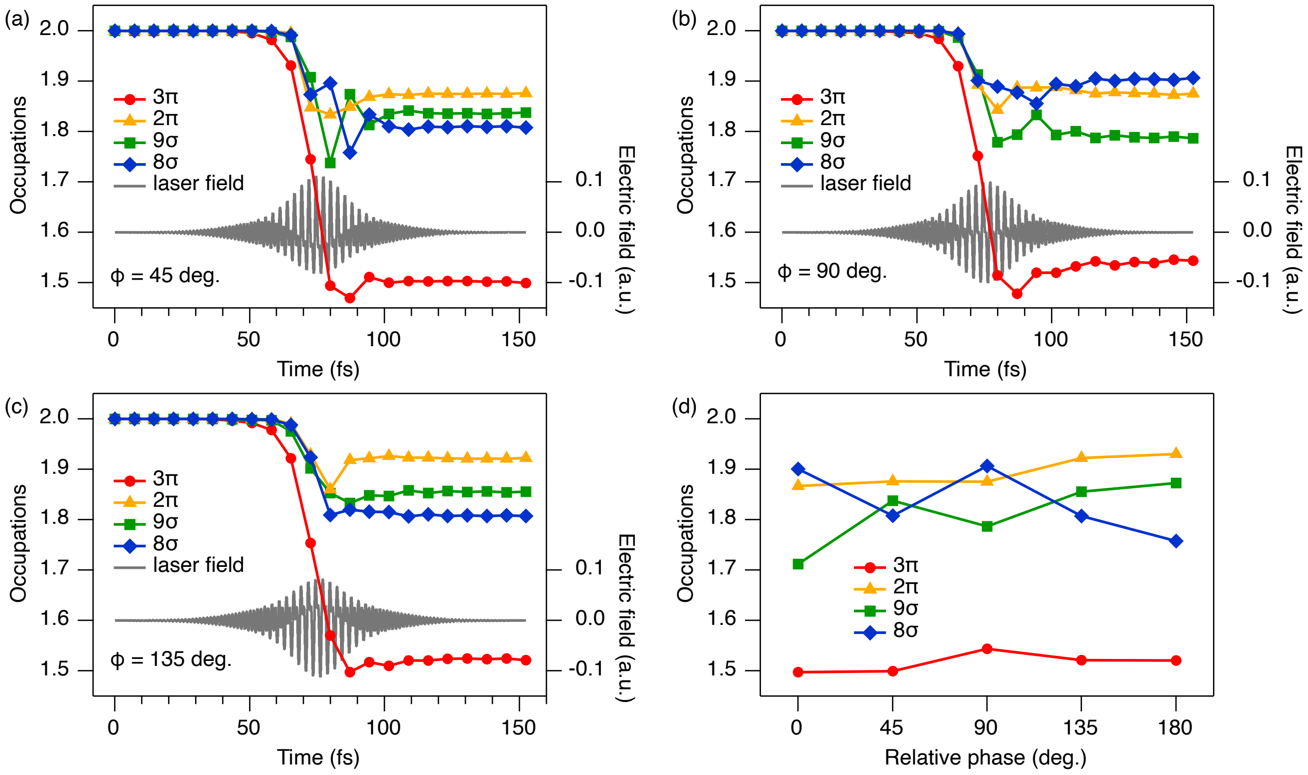

Figure S1. Results of the rtTDDFT calculations. (a) Time-dependent orbital occupations for phase ϕ=45°, (b) same for ϕ=90°, (c) same for ϕ=135°. The shape of laser electric field at each phase is shown as a grey line. (d) Orbital occupations at the end of the simulation for different phases ranging from 0° to 180°, as labeled.

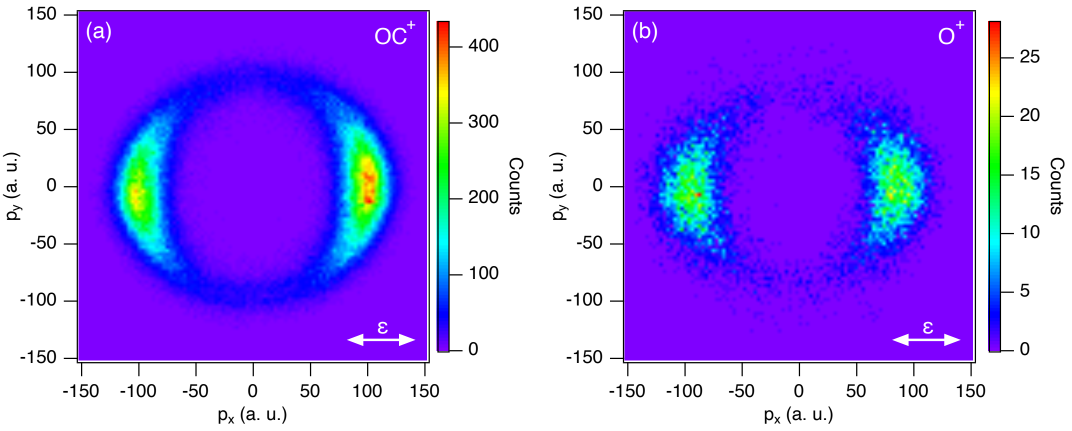

Figure S2. Phase-averaged momentum images of (a) OC^+ fragment ions in the $OC^+ + S^+$ channel and (b) O^+ fragment ions in the $O^+ + CS^+$ channel in the phase-locked two-color laser fields ($I_{\omega+2\omega} = 2 \times 10^{14}$ W/cm^2, $\alpha = 0.14$). The laser polarization direction is denoted as ε.

Regarding the accuracy, within the described level of theory the results are converged and the difference of 11% in n_{emit} is significantly larger than the errors one would expect due to convergence issues with applied rtTDDFT simulations of more than 150 fs, that are state-of-the-art in this field.

2 MOMENTUM IMAGE

The phase-averaged momentum images of the OC^+ and O^+ fragment ions on the xy-plane are shown in Fig. S2. The total laser field intensity is 2×10^{14} W/cm^2 and the intensity ratio is 0.14. Both of the OC^+ and O^+ ions show strong anisotropy and are preferentially ejected to laser polarization direction (horizontal direction) as mentioned in the main text. The momentum images of counter ions (S^+ or CS^+) are mirror images about the origin because we employed coincidence detection technique. Strong anisotropy of momentum images indicate that the dissociation processes for both channels are fast enough to reflects the orientation of parent ions to the fragment ejection direction.

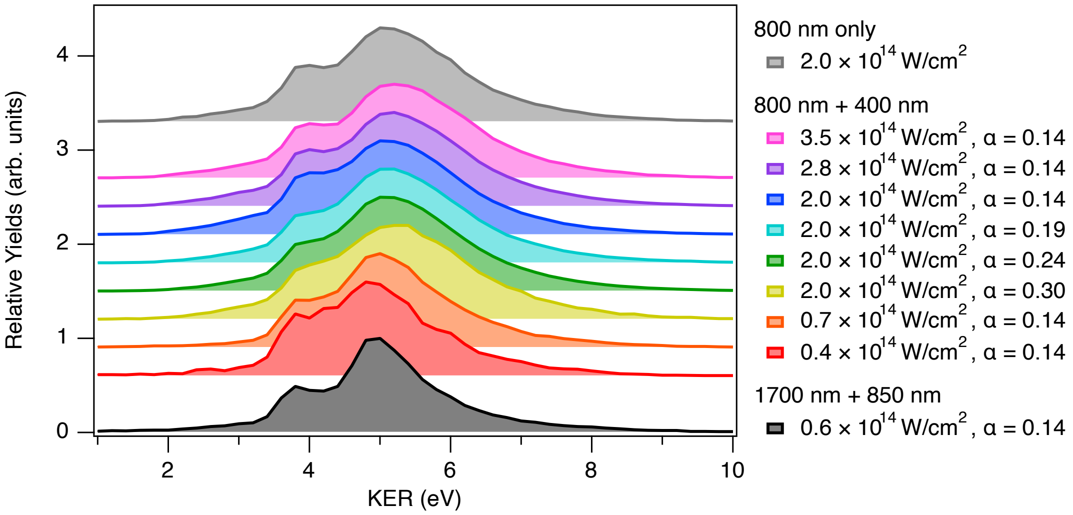

Figure S3. Phase-averaged KER spectra of the $OC^+ + S^+$ channel at different experimental conditions.

3 KER SPECTRA

The phase-averaged KER spectra of the OC^+ + S^+ channel at different experimental conditions are shown in Fig. S3. Note that in contrast to Fig. 3 of the main paper, the KER shown in Fig. S3 is obtained by considering all the fragments (angular range of 90 deg.) to improve the statistics, especially in lower intensity conditions. This broader angular range causes the shoulder structure to become more prominent compared to Fig. 3, indicating that the angular distribution of the shoulder component is broader than the angular distribution of the main peak. This is consistent with our argument that the shoulder structure appears as a result of post-ionization interaction.

The similar structures with main and shoulder peaks are observed regardless of the laser field intensity, intensity ratio, and wavelength. However, as mentioned in the main text, the position of the main peak around 5 eV is shifted towards larger KER direction as the total laser field intensity increases.

4 ASYMMETRY PARAMETER

4.1 Intensity dependence

The total peak intensity dependence of the amplitude of asymmetry parameter at a fixed intensity ratio $\alpha = 0.14$ is shown in Fig. S4. The integrated asymmetry parameters over 5-10 eV for the OC^+ + S^+ channel and 3.5-7 eV for the O^+ + CS^+ channel are fit by a cosine function to evaluate the amplitude. Both of the asymmetry amplitudes of the OC^+ + S^+ and O^+ + CS^+ channels decrease as the total peak intensity increases. This can be understood by a saturation of the ionization probability as observed in the case of HCl (Li et al., 2016). At lower intensities, stronger amplitude side of two-color laser fields mainly contributes to tunneling ionization because tunneling ionization is a highly non-linear process. In the case of polar molecules, tunneling ionization rate shows strong asymmetry reflecting the shape of the outermost molecular orbital and dipole moment as discussed in the main text. Therefore, the parent ions (OCS^+ or OCS^{2+}) are strongly oriented with respect to the laser polarization direction in two-color laser fields, and the fragment ions are preferentially ejected to the oriented direction of the parent ions by prompt dissociation reactions. On the other hand, at higher intensities, the contribution of a weaker amplitude side of two-color laser fields cannot be negligible due to a saturation of tunneling ionization rate as predicted by tunneling ionization theory such as ADK and tunneling ionization rate becomes isotropic. This weakens the orientation degrees of the parent ion and reduces the asymmetry of the fragment ion.

4.2 Ratio dependence

The amplitudes of the asymmetry parameter as a function of α at a fixed peak intensity of 2×10^{14} W/cm^2 are shown in Fig. S5. The asymmetry of the two-color laser electric field amplitude becomes maximum at $\alpha = 0.25$, where also the asymmetry of tunneling ionization becomes the most prominent, and decreases by changing α. Both of the asymmetry amplitudes of the OC^+ + S^+ and O^+ + CS^+ channel showed an unintuitive dependence on α. The amplitudes have maximal values at $\alpha = 0.19$, but oscillates by changing α. Since there are several electronic states within a photon energy of the fundamental pulses (1.55 eV), these states can interact with each other in intense laser fields during dissociation as supported by the rtTDDFT calculations. Strong light-matter interaction can change the asymmetry. For example, the average of cubic electric field $\langle F(t)^3 \rangle$ is important for the nuclear dynamics (potential deformation) (Sato et al., 2003) while the asymmetry of electric field is important for the ionization process. In addition, the electron rescattering excitation would lead OCS^{2+} into different electronic excited states depending on the shape of the laser electric field. For more discussion, detailed calculation including the excitation and dissociation processes is necessary, however competition between several mechanisms such as the phase dependence of ionization step as discussed above, potential deformation, and electron rescattering excitation may change

α dependence of the asymmetry amplitude. The oscillatory behavior of α indicates the importance of not only tunneling ionization but also post-ionization interaction between polar molecules and intense laser fields during the dissociation process.

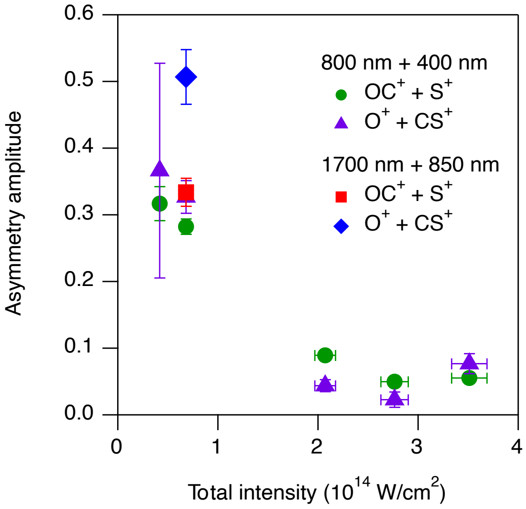

Figure S4. Amplitudes of the asymmetry parameter as a function of the total peak intensity $I_{\omega+2\omega}$ at fixed $\alpha = 0.14$. The OC^+ + S^+ integrated over 5-10 eV (green circles), and O^+ + CS^+ channel integrated over 3.5-7 eV (purple triangles) observed in the 800 nm + 400 nm laser fields. The OC^+ + S^+ channel integrated over 5-10 eV (red squares), and the O^+ + CS^+ channel integrated over 3.5-7 eV (blue diamonds) observed in the 1700 nm + 850 nm laser fields.

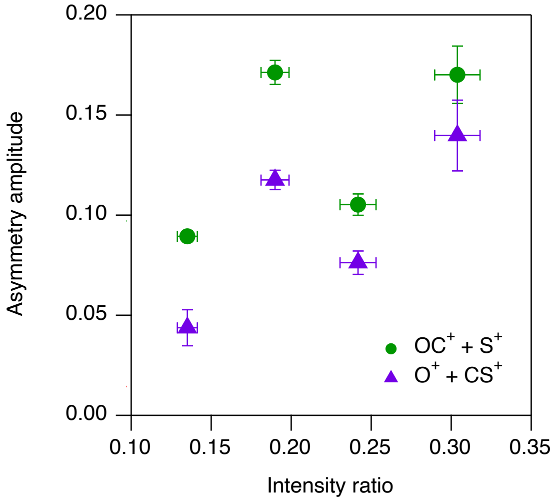

Figure S5. Amplitudes of the asymmetry parameter as a function of the ratio α at the fixed total peak intensity of 2×10^{14} W/cm^2. The OC^+ + S^+ dissociation channel integrated over 5-10 eV (green circles), and the O^+ + CS^+ dissociation channel integrated over 3.5-7 eV (purple triangles).

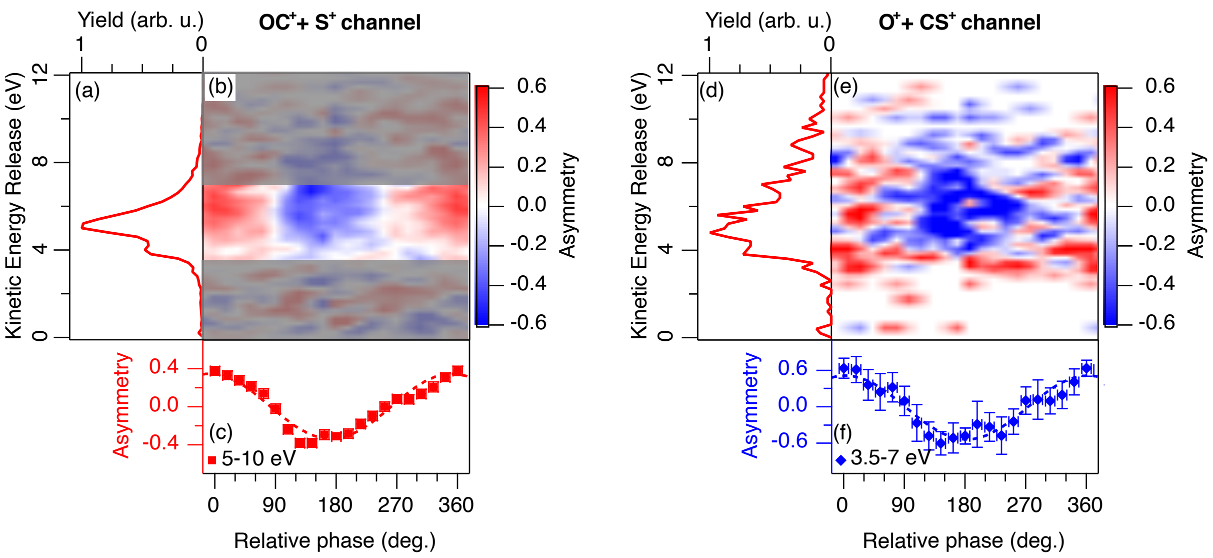

Figure S6. Same as Figure 2 in the main text, but observed in the longer wavelength (1700 nm + 850 nm) two-color laser fields. The total kinetic energy release E_{kin} spectrum of (a) OC^+ + S^+, and (d) O^+ + CS^+. (b), (e) Two-dimensional plot of the asymmetry parameter corresponding to (a) and (d). The errors of asymmetry in grey hatched area in (b) are larger than 0.25, but the errors of asymmetry in all area in (e) are larger than 0.25. (c) Asymmetry parameters of (c) the OC^+ + S^+ channel integrated over 5-10 eV (red squares), and (f) the O^+ + CS^+ channel integrated over 3.5-7 eV (blue diamonds).

4.3 Wavelength dependence

We have also changed the fundamental wavelength from 800 nm to 1700 nm, which is generated by using an optical parametric amplifier. The experimental setup is common as described above, but optical components are replaced for 1700 nm pulses and its second harmonic (850 nm) pulses. The peak intensity at the focal spot is estimated to be 0.6×10^{14} W/cm^2, and the ratio α is set to 0.14 to compare with the results of 800 nm. The pulse durations are measured to be 100 fs by SHG-FROG. The KER spectra of OC^+ + S^+ and O^+ + CS^+ channels are shown in Figs. S6(a) and (d). The KER spectrum of OC^+ + S^+ channel generated in the longer wavelength laser fields shows the similar peak structure with that observed in the 800 nm laser fields. The main peak is slightly shifted from 5.2 eV to 5.0 eV. It would be the effect of the enhanced ionization at the longer internuclear distance because of weaker intensity and longer wavelength than those utilized in the main text. In addition, the peak structure is more significant than that observed with 800 nm laser fields. This may be also due to weaker intensity and longer wavelength. Since weaker intensity suppress the population in the excited state in dication, the dissociation from the ground state would become prominent. The longer wavelength provides potential deformation for longer time, so the dissociation from the bound state could be enhanced.

The amplitude of the asymmetry parameter of OC^+ + S^+ channel in the 1700 nm laser fields is higher than that in the 800 nm laser fields in spite of the same intensity and ratio (see Fig. S4 and Figs. S6(c) and (f)). In longer wavelength fields, tunneling ionization process becomes more prominent than in shorter wavelength fields because other ionization process, multiphoton ionization, is suppressed due to the lower photon energy. On the other hand, both of the OC^+ + S^+ and O^+ + CS^+ channels show no kinetic energy dependence as shown in Figs. S6(b) and (e). Since the coupling between electronic states would be also suppressed in the longer wavelength fields because of a lack of the photon energy, the wavelength dependence also suggests the important roles of post-ionization interactions for the fragmentation process of

polar molecules. This indicates that several mechanisms such as electron recollisional excitation, potential deformation, and population transfer can be contributed to the appearance of this shoulder peak.

REFERENCES

Li, H., Tong, X. M., Schirmel, N., Urbasch, G., Betsch, K. J., Zherebtsov, S., et al. (2016). Intensity dependence of the dissociative ionization of DCl in few-cycle laser fields. *J. Phys. B At. Mol. Opt. Phys.* 49, 015601. doi:10.1088/0953-4075/49/1/015601

Sato, Y., Kono, H., Koseki, S., and Fujimura, Y. (2003). Description of molecular dynamics in intense laser fields by the time-dependent adiabatic state approach: Application to simultaneous two-bond dissociation of co_2 and its control. *Journal of the American Chemical Society* 125, 8019–8031. doi:10.1021/ja0344819. PMID: 12823025
